# Design and optimisation of rapid real‐time PCR assays for the detection of key *C* *ulicoides* species

**DOI:** 10.1111/mve.70088

**Published:** 2026-06-19

**Authors:** Elsie Isiye, Angela Valcarcel Olmeda, Thomas Curran, David O'Neill, Theo De Waal, Gerald Barry, Aidan O'Hanlon, James O'Shaughnessy, Marion England, Annetta Zintl, Denise O'Meara

**Affiliations:** ^1^ SETU School of Science and Computing, South East Technological University Waterford Ireland; ^2^ UCD School of Veterinary Medicine, University College Dublin Dublin 4 Ireland; ^3^ National Museum of Ireland‐Natural History Dublin 2 Ireland; ^4^ Central Veterinary Research Laboratory, Backweston Laboratory Campus Celbridge Ireland; ^5^ The Pirbright Institute Surrey UK

**Keywords:** *Culicoides*, ITS2 gene region, real‐time PCR, species‐specific primers

## Abstract

In extensive surveillance programmes of *Culicoides* biting midges (Diptera: Ceratopogonidae), morphological identification can be time‐consuming and difficult, while DNA barcoding, although highly accurate, may not be cost‐effective or suitable for rapid analysis, as it requires individual specimen processing. To address these limitations, we developed a rapid screening method using real‐time PCR assays with either SYBR Green or hydrolysis probe‐based detection chemistries. Species‐specific primers and, where necessary, hydrolysis probes were designed based on the updated sequences of the ITS2 region of seven *Culicoides* species. The specificity and efficiency of these assays were validated both in silico and through real‐time PCR testing on target and non‐target *Culicoides* species, tested individually and as mixed‐species samples. The new real‐time PCR assays detect the presence of key species including *C. obsoletus*, *C. scoticus*, *C. chiopterus*, *C. dewulfi*, *C. pulicaris*, *C. punctatus* and *C. impunctatus* in pools of specimens. The molecular techniques developed in this study provide a valuable tool for accurate and high‐throughput *Culicoides* surveillance, which can be used for year‐round monitoring of adult midges in traps, larvae and in environmental samples, potentially revealing novel insights into the spatial and temporal turnover of *Culicoides* species. These methods can be applied to large‐scale vector screening programmes involving pooled samples, addressing the limitations of previously described methods used in midge surveillance.

## Graphical abstract

- Species‐specific real‐time PCR assays were designed and optimised using primer/probe sets targeting *C. obsoletus*, *C. scoticus*, *C. chiopterus*, *C. dewulfi*, *C. pulicaris*, *C. punctatus* and *C. impunctatus*.
- Primer specificity and amplification efficiency were validated through in silico specificity analysis and real‐time PCR amplification profiling.
- The novel assays were evaluated using pooled field‐trap samples to assess their suitability for high‐throughput screening of target species and they enabled their rapid and accurate detection.

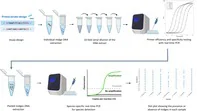

## INTRODUCTION

In Europe, *Culicoides* biting midges (Diptera: Ceratopogonidae) are well recognised as both disease vectors and nuisance pests (Carpenter et al., 2013; Prudhomme et al., 2025). Commonly occurring *Culicoides* species in Ireland include *Culicoides obsoletus* Meigen, 1818, *Culicoides scoticus* Downes & Kettle, 1952, *Culicoides dewulfi* Goetghebuer, 1936, *Culicoides chiopterus* Meigen, 1830, *Culicoides pulicaris* and *Culicoides punctatus* Meigen, 1804. These species are of considerable veterinary importance due to their role as vectors of bluetongue virus (BTV) and Schmallenberg virus (SBV), both of which have significant impacts on livestock health and productivity (Collins et al., 2018; Isiye et al., 2025; Jess et al., 2018; McCarthy et al., 2010). In addition, *Culicoides impunctatus* Goetghebuer, 1920 is widely distributed and recognised as an important nuisance species. Entomological studies have also shown that these vector‐competent species dominate Irish midge populations, collectively accounting for over 90% of specimens identified (Collins et al., 2018). Their clinical importance is underscored by the introduction and circulation of arboviruses in Ireland. For example, SBV was first detected in Northern Ireland in January 2012 and subsequently in the Republic of Ireland (ROI) in October 2012, followed by widespread circulation in the following years (Bradshaw et al., 2012; Barrett et al. 2015; Collins et al., 2019). More recently, BTV was detected in Northern Ireland in November 2025 and in the ROI in January 2026 (Gov.UK 2025; NDCC Bluetongue Virus Update 1 of 2026). These events highlight the capacity for arbovirus emergence and re‐emergence in Ireland in the presence of competent vector populations and indicate a clear need for enhanced surveillance approaches. Such systems should combine high specificity and sensitivity with the capacity to detect target species within pooled light‐trap samples, enabling rapid and large‐scale national monitoring.

Traditionally, the surveillance of these species has relied on morphological characteristics, including wing patterns, head structures and genitalia (Mathieu et al., 2012), a method that has served as the foundation for the identification and classification of insect species. DNA barcoding of mitochondrial and ribosomal gene regions has been increasingly used, providing high accuracy in distinguishing closely related species (Mathieu et al., 2020; Mignotte et al., 2020) and identifying new species (Nielsen et al., 2015; Sarvašová et al., 2017). Despite these advantages, both approaches remain labour‐intensive and costly in large‐scale vector surveillance programmes, as they require the processing of very high numbers of individual specimens as well as specialised expertise for accurate species‐level identification. On the other hand, DNA metabarcoding, although capable of detecting a broad range of species in complex mixtures or pooled samples (Wu et al., 2025), often identifies numerous non‐target organisms and depends on extensive reference databases, which are still limited for groups such as *Culicoides* midges.

While genus‐specific primers have been successfully used to distinguish *Culicoides* from other Ceratopogonidae (Cêtre‐Sossah et al., 2004), species‐specific tests provide a robust approach for simplifying their identification and allowing targeted surveillance. The first conventional species‐specific PCR tests to be developed targeted *Culicoides imicola* Kieffer, 1913, to enable its detection in insect pools (Casati et al., 2009; Cêtre‐Sossah et al., 2004) as it was considered to be the primary vector of BTV in southern Europe (Carpenter et al., 2009; Mellor & Boorman, 1995; Miranda et al., 2003). However, as BTV spread further north and SBV emerged beyond the range of *C. imicola* (Gloster et al., 2008; Carpenter et al., 2009, 2013; Wilson & Mellor, 2009), there was an expansion in recognised vectors leading to the development of additional species‐specific conventional PCR assays (Lehmann et al., 2012; Mathieu et al., 2011; Nolan et al., 2007; Pagès et al., 2009; Stephan et al., 2009).

Real‐time PCR of *Culicoides* spp. was similarly applied to offer a more sensitive and specific method, with its initial utilisation of the internal transcriber spacer 1 (ITS‐1) gene region of *C. imicola* sensu stricto. This significantly improved the surveillance of this species in France, as it enabled detection of the target species in mixed samples (Cêtre‐Sossah et al., 2008). Subsequently, a quantitative duplex real‐time TaqMan PCR assay was developed to determine the relative abundances of *C. obsoletus* and *C. scoticus* in pooled samples (Mathieu et al., 2011). Concurrently, Wenk et al. (2012) designed real‐time PCR assays targeting the mitochondrial cytochrome c oxidase subunit 1 (CO1) gene for the specific identification of both vector and non‐vector *Culicoides* species; however, cross‐species reactivity was observed between *C. chiopterus*, *C. scoticus*, *C. deltus*, *C. grisescens* and *C. obsoletus*. Dähn et al. (2025) later developed quantitative real‐time PCR assays targeting six members of the *Culicoides* subgenus *Avaritia*; however, the study highlighted cross‐reactivity in the multiplex format, which was attributed to overlapping primer‐probe interactions. The presence of multiple haplotypes within different *Culicoides* species represents significant genetic diversity and presents a challenge for species‐specific assay design, as certain haplotypes may be excluded from detection or may lead to cross‐reactivity when degenerate primers are used to accommodate sequence variation (Wenk et al., 2012). These limitations are particularly problematic in the context of unsorted, field‐collected specimens, where accurate and high‐throughput species identification is essential.

In Ireland, morphological and molecular characterisation efforts have contributed to the development and validation of the Irish *Culicoides* species list (Collins et al., 2018; Isiye et al., 2025; McCarthy et al., 2010). However, species‐specific molecular assays targeting key vector taxa remain limited. Accordingly, this study aimed to develop and validate a high‐throughput species‐specific real‐time PCR assay targeting the internal transcribed spacer 2 (ITS2) region for the detection of these economically and epidemiologically important *Culicoides* species in light‐trap collections. Species‐specific assays targeting key vector and nuisance species (*C. obsoletus*, *C. scoticus*, *C. chiopterus*, *C. dewulfi*, *C. pulicaris*, *C. punctatus* and *C. impunctatus*) were developed. This approach addresses the limitations associated with morphological identification and broad‐range molecular methods, providing a scalable and sensitive tool for enhancing surveillance.

## MATERIALS AND METHODS

### Primer and probe design

A nucleotide BLAST (BLASTn) search was performed using the ITS sequences obtained from seven *Culicoides* biting midge species: *C. obsoletus*, *C. scoticus*, *C. chiopterus*, *C. dewulfi*, *C. pulicaris*, *C. punctatus* and *C. impunctatus*, which were generated through DNA barcoding analysis by Isiye et al. (2025). The corresponding reference sequences from GenBank with the highest similarity to the Irish *Culicoides* sequences were selected, and multiple sequence alignment (MSA) was performed using the EMBL‐EBI Clustal Omega program (Madeira et al., 2022). The alignment included the following sequences: *C. obsoletus* (MK893033), *C. scoticus* (MK893045), *C. chiopterus* (MK893002), *C. dewulfi* (MK893005), *C. pulicaris* (DQ371264), *C. punctatus* (DQ371247) and *C. impunctatus* (EU908207) (Gomulski et al., 2006; Mathieu et al., 2020). Conserved and variable regions were identified within the alignment, and the forward primer, reverse primer, and for certain tests, a probe was designed based on interspecies variable regions to ensure test specificity, using Primer Express™ Software v3.0.1. Primer specificity was evaluated in silico using the Primer‐BLAST software (Ye et al., 2012), publicly available at http://www.ncbi.nlm.nih.gov/tools/primer-blast.

### Efficiency testing

The efficiency of the novel real‐time PCR primers was evaluated using a 5‐log dilution series of target species DNA obtained from a previous study by Isiye et al. (2025). Each reaction consisted of 5 μL of 2× FastStart Universal SYBR Green Mastermix (ROX) (Sigma‐Aldrich), 0.4 μL of forward and reverse primer mix with a final concentration of 200 nM, 3.6 μL of molecular‐grade water and 1 μL of extracted DNA as the template. The negative control reactions included 1 μL of molecular‐grade water instead of the DNA template. Reactions were prepared in a final volume of 10 μL and performed in triplicate using a QuantStudio™ 3 Real‐Time PCR System (Applied Biosystems). The real‐time PCR profile consisted of an initial incubation at 50°C for 2 min, followed by denaturation at 95°C for 10 min, and 40 cycles of 95°C for 15 s and 60°C for 1 min. Primer specificity and sensitivity were assessed using amplification and melt curve analyses. A standard curve was generated, and the primer efficiency (*E*) was calculated, with the recommended efficiency range being 90%–110%. The curve was visualised using the ggplot2 package (Wickham et al. 2023) in R version 4.4.1 (R Core Team, 2025).

### Cross‐species reactivity testing

To determine cross‐species amplification, all primer sets were tested against 0.25 ng DNA of each non‐target species, as described above. All assays were first tested as SYBR‐based assays; however, for assays that amplified the target and non‐target species, a probe was introduced to increase the specificity of the tests. Each probe‐based reaction contained 5 μL TaqMan™ Fast Advanced Master Mix (Applied Biosystems), 0.2 μL of forward and reverse primers, probe mix with a final concentration of 250 nM, 3.8 μL of molecular‐grade water and 1 μL of extracted DNA. Negative controls were prepared by replacing the DNA template with 1 μL molecular‐grade water.

### Application to mixed field trap samples

*Culicoides* specimens were collected in June 2023 from 13 field sites across Ireland using light traps. The sites were distributed across multiple counties, including Dublin (OVI 1), Meath (OVI 2), Laois (OVI 3), Cork (OVI 4), Kerry (OVI 5), Carlow (OVI 6 and 7), Galway (OVI 8), Clare (OVI 9), Limerick (OVI 10), Mayo (OVI 11), Waterford (OVI 12) and Kilkenny (OVI 13), providing broad geographic coverage across the country. Specimens were morphologically classified (Valcarcel et al. in preparation) and subsequently pooled by field site, resulting in 13 composite samples. Since specimens from each pool reflected natural trap collections, pool sizes varied between sites. The seven target species included in this study represent the most commonly occurring and epidemiologically important *Culicoides* species in Ireland. *Culicoides* specimens preserved in ethanol were first air‐dried for approximately 1 h to remove residual ethanol. Pooled samples were then manually homogenised in 1.5 mL microcentrifuge tubes using a sterile pipette tip in the presence of extraction buffer. DNA was extracted using the NucleoSpin DNA Extraction Kit (Macherey‐Nagel), following the manufacturer's protocol, and eluted in 100 μL of elution buffer. DNA concentration and purity were assessed using a NanoDrop™ 8000 spectrophotometer (Thermo Scientific). Each extracted DNA sample was subjected to a 1:10 dilution and used as template DNA. All reactions were performed in triplicate for both SYBR‐based and hydrolysis probe‐based assays. The reaction conditions followed the respective protocols previously established during cross‐species reactivity testing, and the average Ct values and melting temperatures (Tm) were recorded. To visualise the presence or absence of each species within the mixed samples, a dot plot was generated in R version 4.4.1 (R Core Team, 2025) using the ggplot2 package (Wickham et al. 2023).

## RESULTS

### Primer and probe design

Seven real‐time PCR primer sets were designed targeting the *Culicoides* species: *C. pulicaris*, *C. scoticus*, *C. dewulfi*, *C. chiopterus*, *C. obsoletus*, *C. punctatus* and *C. impunctatus* (Table 1).

**TABLE 1:** Primers and probes designed for the specific detection of *Culicoides* species, along with the standard curve results and efficiencies for the species‐specific primer sets selected for each target species.

Species	Primer/probe (5′ to 3′)	Slope	*R* ^2^	Efficiency	Amplicon length (bp)
*C. chiopterus*	CCHIOF‐AAACTATCTTATGTGTTAACGCGCA	−3.49	0.999	93.434	102
	CCHIOR‐GCACAAGTACCGTTAACCATATCCTT				
*C. pulicaris*	CPULF‐AGGTCTGTGTGGGGTACACATG	−3.344	0.99	99.103	103
	CPULR‐CCATATCATCATAGCATACACTTAGAGGT				
*C. dewulfi*	CDEWF‐ACAGAGTCTGTGTGGGGTACACAT	−3.391	0.998	97.192	90
	CDEWR‐GCACTTATAATGCCACATAACTGACAC				
*C. impunctatus*	CIMPF‐GTGTGTTTGAATAAACATTGTAAGTGCA	−3.448	0.997	94.999	192
	CIMPR‐GGATGGTATGTTAAGGTAAGTAACAAAAACTAT				
*C. scoticus*	CSCOF‐GCATGATGTGATCATATGGATGTAAC	−3.387	0.996	97.357	149
	CSCOR‐AGTTTACTAAATGCAGAGCACATTTGA				
*C. punctatus*	CPUNF‐GGTACACATGGTTGAGTGTCGTTAT	−3.459	0.996	94.57	141–143
	CPUNR‐GCTTAATATATTTGTTACCACCACACTT				
	FAM‐GTGTTTGAAAGAACATTTAAGTG‐MGBEQ				
*C. obsoletus*	COBSOF‐TCTTATGTGTCTTTAACGGGCATC	−3.512	0.998	92.647	105
	COBSOR‐TGCACACAAAGTAGCGTTAACCAT				
	FAM‐TGTGATCATATGGATGTAACA‐MGBEQ				

### Efficiency testing

When tested on the bench, the selected primer sets exhibited a well‐defined dynamic range of amplification, producing five distinct amplification curves (Figures 1 and 2). Melt curve analysis further validated the specificity of amplification by generating distinct peaks at specific melting temperatures (*T*_m_) (Figure S1). Additionally, standard curve analysis (Figures 1 and 2) confirmed that the primer efficiency remained within the optimal range of 90%–100% (Table 1 and Figure S2).

**FIGURE 1:**
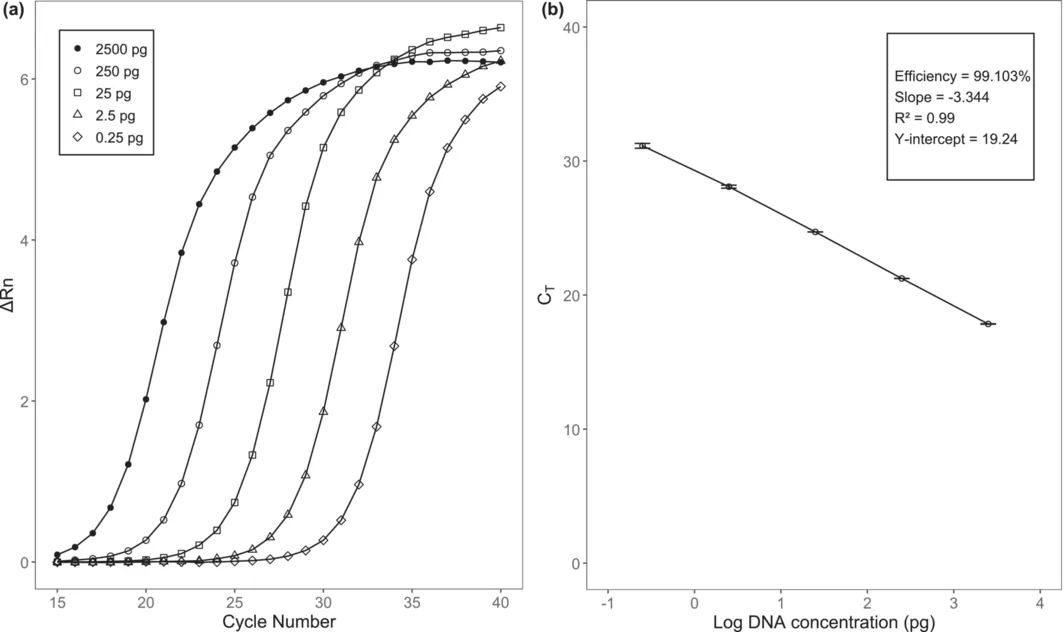
Amplification of *Culicoides pulicaris* target DNA using CPUL primers. (a) Amplification plot showing a 1:10 serial dilution series with corresponding amplification curves and (b) standard curve showing the linear regression of Ct values against DNA concentration, demonstrating assay efficiency and sensitivity.

**FIGURE 2:**
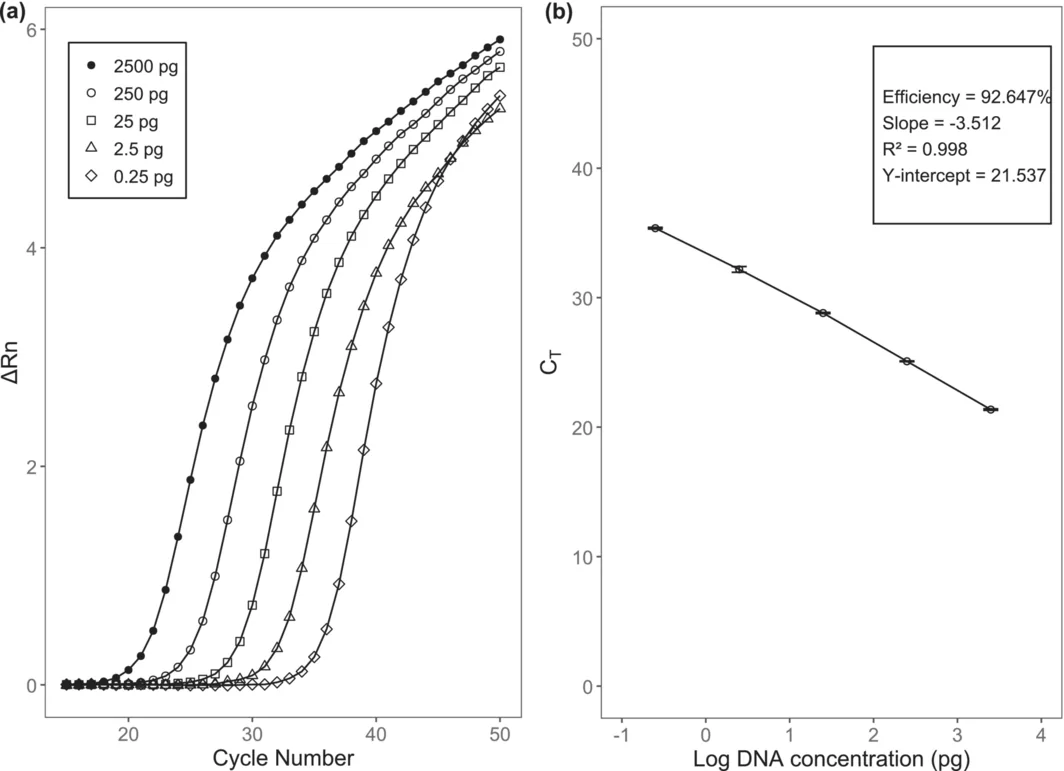
Amplification of *Culicoides obsoletus* target DNA using COBSO primers and a TaqMan® probe. (a) Amplification plot displaying a 1:10 serial dilution series with corresponding amplification curves and (b) Standard curve showing the linear regression of Ct values against DNA concentration, demonstrating assay efficiency and sensitivity.

### Cross‐species reactivity testing

As shown for *C. chiopterus* in Figure 3, the assays effectively differentiated the target species while minimising non‐specific amplification. Setting a cut‐off Ct value of 34 ensured that false positives were not included, as any amplification beyond this value was not considered indicative of target detection. Furthermore, no cross‐reactivity was detected in the assays developed for *C. pulicaris* (CPUL), *C. impunctatus* (CIMP), *C. scoticus* (CSCO) and *C. dewulfi* (CDEW). The designed SYBR‐based assays lacked specificity for two species. These included *C. punctatus*, which was successfully amplified using the primers CPUNF and CPUNR at a Ct of 25. However, *C. impunctatus* was also amplified (at a Ct of 30), indicating some cross‐reactivity. To enhance specificity, a probe was introduced, which improved the assay by amplifying *C. punctatus* at a Ct value of 23 while eliminating cross‐reactivity with *C. impunctatus*. The second species was *C. obsoletus*, which was successfully amplified using COBSOF and COBSOR with a Ct of 21 and Tm of 71°C. However, non‐target species, including C. *pulicaris*, *C. chiopterus*, *C. dewulfi*, *C. impunctatus*, *C. scoticus* and *C. punctatus* were also amplified, though at Ct values >34. To improve specificity, a probe was introduced to eliminate non‐target amplification, and the resulting Ct values are presented in Table 2. During cross‐species reactivity testing of the *C. obsoletus* assay, occasional late amplification was observed at Ct values ranging from 35.6 to 38.6. These amplifications were inconsistent, occurring in only one or two of the three technical replicates, and were detected in some non‐target species despite the inclusion of a species‐specific probe, which reduced but did not fully eliminate these non‐specific signals. No amplification was observed in any of the no‐template control (NTC) assays. The average Ct and Tm values for all the assays are presented in Table 2.

**FIGURE 3:**
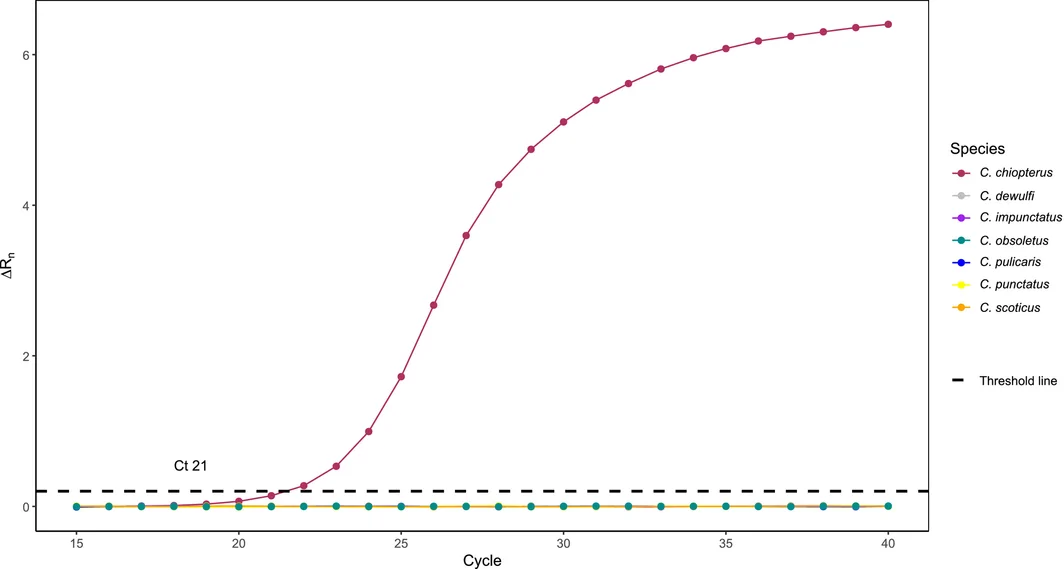
Amplification of *Culicoides chiopterus* and non‐target species using species‐specific primers CCHIO, ensuring selective amplification without cross‐reactivity.

**TABLE 2:** The average cycle threshold (Ct) and melting temperature (Tm) values for each species‐specific assay using SYBR Green‐based and probe‐based real‐time PCR. Note: Tm values are not reported for *C. obsoletus* and *C. punctatus* because probe‐based assays do not utilise melting temperature analysis for target detection.

Assay name	Species	Average Ct	Average Tm (°C)	Standard deviation
CCHIO	*C. chiopterus*	21	69	0.07
CPUL	*C. pulicaris*	21	70	0.09
CDEW	*C. dewulfi*	23	70	0.06
CIMP	*C. impunctatus*	27	68	0.48
CCSO	*C. scoticus*	26	69	0.07
CPUN	*C. punctatus*	23	‐	0.21
COBSO	*C. obsoletus*	22	‐	0.07

### Application to mixed field trap samples

A total of 840 *Culicoides* specimens were collected and pooled into 13 samples, each representing a distinct field site (OVI 1–OVI 13). The number of midges per pool ranged from 21 to 96 individuals (Table 3).

**TABLE 3:** Pooled *Culicoides* field samples, including site identifiers, sample IDs, pool sizes and species groups detected using the species‐specific real‐time PCR assays.

Site ID	Sample ID	No. of midges per pool	Species detected in pool using real‐time PCR
OVI 1	p1	21	*C. chiopterus, C. dewulfi, C. scoticus, C. obsoletus*
OVI 2	p2	60	*C. chiopterus, C. pulicaris, C. dewulfi, C. scoticus, C. puntcatus, C. obsoletus*
OVI 3	p3	32	*C. chiopterus, C. pulicaris, C. scoticus, C. puntcatus, C. obsoletus*
OVI 4	p4	92	*C. chiopterus, C. pulicaris, C. dewulfi, C. scoticus, C. obsoletus*
OVI 5	p5	89	*C. chiopterus, C. pulicaris, C. dewulfi, C. impunctatus, C. scoticus, C. puntcatus, C. obsoletus*
OVI 6	p6	88	*C. chiopterus, C. pulicaris, C. dewulfi, C. impunctatus, C. scoticus, C. puntcatus, C. obsoletus*
OVI 7	p7	75	*C. chiopterus, C. pulicaris, C. dewulfi, C. scoticus, C. obsoletus*
OVI 8	p8	94	*C. chiopterus, C. pulicaris, C. dewulfi, C. impunctatus, C. scoticus, C. puntcatus, C. obsoletus*
OVI 9	p9	41	*C. chiopterus, C. pulicaris, C. dewulfi, C. impunctatus, C. scoticus, C. puntcatus, C. obsoletus*
OVI 10	p10	96	*C. chiopterus, C. pulicaris, C. dewulfi, C. impunctatus, C. scoticus, C. puntcatus, C. obsoletus*
OVI 11	p11	37	*C. chiopterus, C. pulicaris, C. dewulfi, C. impunctatus, C. scoticus, C. puntcatus, C. obsoletus*
OVI 12	p12	22	*C. chiopterus, C. pulicaris, C. dewulfi, C. scoticus, C. obsoletus*
OVI 13	p13	93	*C. chiopterus, C. pulicaris, C. dewulfi, C. obsoletus*

DNA concentrations obtained from pooled extractions ranged from 3.0 to 27.3 ng/μL. A presence/absence dot plot (Figure 4) summarising the detection of target *Culicoides* species across the 13 pooled field samples showed that *C. obsoletus* and *C. chiopterus* were detected in all samples. *Culicoides dewulfi*, *C. scoticus* and *C. pulicaris* were each detected in all but one sample (p3, p13 and p1, respectively). *Culicoides punctatus* was absent from five samples (p1, p4, p7, p12 and p13), while *C. impunctatus* was the least frequently detected species, being absent from six samples (p1, p2, p4, p7, p12 and p13). The corresponding cycle threshold (Ct) values for each assay are provided in Table S3. The Ct values showed a semi‐quantitative relative estimate rather than absolute counts of each species per pool.

**FIGURE 4:**
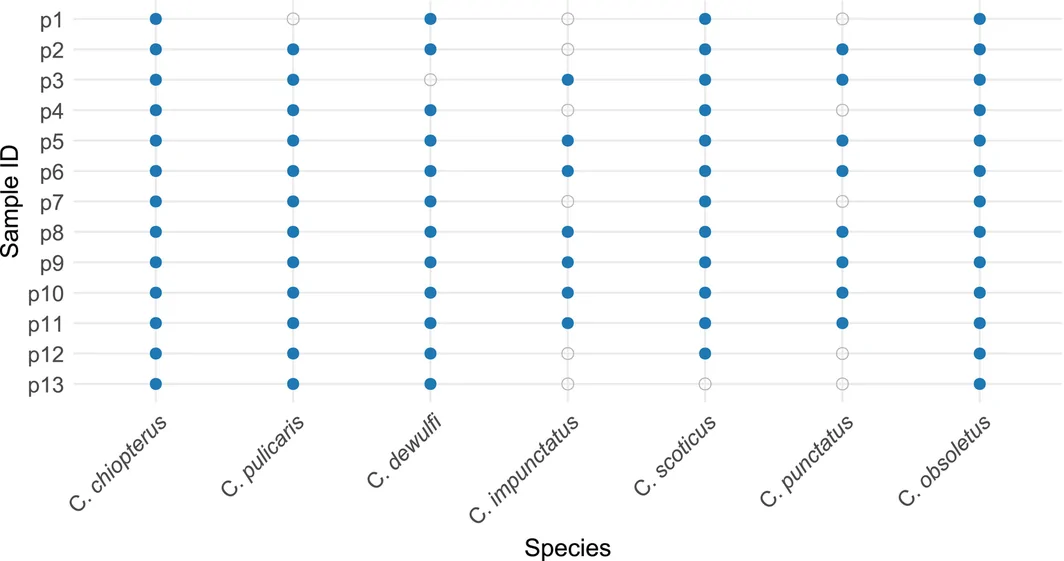
Field validation of novel species‐specific real‐time PCR assays for screening *Culicoides* species. The dot plot illustrates species presence or absence within each pooled field trap sample (p1–p13; *n* = 840).

## DISCUSSION

Advances in molecular techniques have significantly improved biodiversity monitoring, enhancing the efficiency of vector surveillance through accurate species discrimination and genetic comparative analyses across different geographic regions (Mignotte et al., 2021; Onyango et al., 2015; Tay et al., 2016). To aid in molecular surveillance, diagnostic tests have been developed by designing primers that target highly variable regions, ensuring high specificity and amplification efficiency. Tools such as Primer 3 and Primer Express™ Software are used to select primers, considering factors like melting temperature (Tm), primer length, GC content and secondary structure (Rozen & Skaletsky, 2000). Species‐specific real‐time PCR assays are designed for high sensitivity, enabling the detection of a *Culicoides* species within a mixed insect pool. Some assays have demonstrated the ability to detect a single *C. imicola* in a pool of 3200 *Culicoides* or within 65 mg of material from a light trap (Cêtre‐Sossah et al., 2004), while others can detect specific species in pools of up to 1000 *Culicoides* (Wenk et al., 2012). The mitochondrial COI gene has been commonly used in the design of species‐specific assays because of its low intraspecific variability, which accounts for haplotype diversity, and high interspecific variation (Aguilar‐Vega et al., 2021; Balczun et al., 2009; Dallas et al., 2003; Pagès et al., 2009). However, for closely related or cryptic species, the more variable ITS region is often preferred (Curran et al., 2025; Isiye et al., 2025; Matsumoto et al., 2009). The ITS1 region was initially used to develop species‐specific assays for *C. imicola* (Cêtre‐Sossah et al., 2008), while later studies targeted the ITS2 region to design real‐time PCR assays for *C. obsoletus*, *C. scoticus* and *Culicoides montanus* Wirth & Blanton, 1969, demonstrating improved sensitivity compared to conventional assays (Goffredo et al., 2010). The entire ITS1‐5.8S‐ITS2 region has also been used in quantitative duplex real‐time PCR assays for detecting and quantifying *C. obsoletus* and *C. scoticus* (Mathieu et al., 2011). In this study, the ITS2 region was selected for primer design because it has been successfully used for the molecular characterisation of *Culicoides* species in Ireland, confirmed through both morphological and DNA barcoding of individual specimens (Isiye et al., 2025). This provided us with verified, high‐quality sequences and ample genetically scored positive control samples for assay development and validation.

In this study, we developed five SYBR‐based and two probe‐based real‐time PCR protocols for identifying *C. obsoletus, C. scoticus, C. chiopterus, C. dewulfi, C. pulicaris, C. punctatus* and *C. impunctatus*, species commonly found across Ireland (Collins et al., 2018; Isiye et al., 2025; Jess et al., 2018; McCarthy et al., 2010). These species are the most abundant and epidemiologically significant *Culicoides* in Ireland. The intention was not to replace comprehensive species diversity surveys, but to provide a rapid, targeted surveillance tool for key vector species. The assays are highly suitable for large‐scale monitoring where the primary objective is to detect presence/absence of key vector species and to estimate their relative abundance across sites and time. Pre‐sorting of *Culicoides* specimens from bulk trap collections prior to pooling and molecular analysis enabled validation of assay specificity and sensitivity. It is important to point out that while assay design incorporated extensive in silico specificity testing (using Primer‐BLAST) to minimise non‐target amplification of other *Culicoides* as well as other taxa, the assays are theoretically capable of detecting *Culicoides* DNA within mixed‐species samples; we currently recommend sorting *Culicoides* from other insects to maximise assay sensitivity and reliability. Future work will evaluate the performance of these assays on unsorted bulk samples.

Sequence alignments of the seven *Culicoides* species were used to design species‐specific primers, following approaches similar to those described in previous studies (Mathieu et al., 2011; Nolan et al., 2007; Stephan et al., 2009). While primer design aimed to balance specificity and amplification efficiency (Dieffenbach et al., 1993), in silico predictions did not always align with results achieved at the bench; therefore, designing multiple primer pairs enabled the use of alternative primers where amplification was absent or inefficient. For species such as *C. impunctatus*, the reverse primer selected was designed to be longer than most typical real‐time PCR primers to ensure adequate specificity owing to the lack of a potential variable region in the alignment. The primary aim was to design SYBR assays, as they are more economical than TaqMan® probes. While this was successfully achieved for five species (*C. pulicaris*, *C. scoticus*, *C. impunctatus*, *C. dewulfi* and *C. chiopterus*), for *C. obsoletus* and *C. punctatus*, specificity was increased through the inclusion of TaqMan® probes.

Primers were designed to account for haplotype diversity, while degenerate bases were avoided to minimise non‐specific amplification and enhance PCR efficiency. All seven primer sets accounted for haplotype diversity within each *Culicoides* species, as demonstrated by the Primer‐BLAST results. Specifically, CPUL matched eight *C. pulicaris* and five *C. lupicaris* haplotypes; CIMP matched two *C. impunctatus*; CDEW matched 20 *C. dewulfi*; CCHIO matched seven *C. chiopterus*; CPUN matched eight *C. punctatus*; and CSCO matched 14 *C. scoticus*. However, the COBSO primers matched a wider range of haplotypes, including 21 haplotypes of *C. obsoletus*, 15 of *C. montanus*, two of *Culicoides sinanoensis* Tokunga, 1937, and one of *C. scoticus* (Table S4). Further analysis by sequence alignment confirmed that the COBSO probe successfully excluded most non‐target species; however, it could likely bind to one haplotype each of *C. sinanoensis* (MK893047), *C. scoticus* (FN263316) and *C. montanus* (MK893025). Representative specimens for these three specific haplotypes were not available, preventing experimental validation of this limitation. The *C. sinanoensis* haplotype identified in Russia (Mathieu et al., 2020) belongs to the eastern Palearctic *obsoletus* complex. Primer‐BLAST results detected four SNPs, three on the forward primer and one on the reverse primer, potentially increasing the Ct values if amplified. The *C. scoticus* haplotype, deposited by Kiehl et al. (2009), was collected in Germany (2007–2008) after the 2006 BTV outbreak. In their study, this *C. scoticus* haplotype was considered a *C. obsoletus* variant rather than a distinct species. However, recent studies have confirmed it as a distinct cryptic species (Mathieu et al., 2020; Mignotte et al., 2020). Finally, *C. montanus* is a rare species with a limited distribution in the Palearctic region. It has been observed in Morocco, areas with high land surface temperatures in Italy, and in small numbers in France, Norway and Spain (Aguilar‐Vega et al., 2021; Gomulski et al., 2005; Mathieu et al. 2007, Mathieu et al., 2012; Mignotte et al., 2020). Morphologically, *C. montanus* is part of the obsoletus complex and is characterised by a deep sensory pit and hypertrophied trichoid sensilla (hair‐like structures of different lengths) (Mathieu, 2011). Genetically, *C. montanus* and *C. obsoletus* form a monophyletic clade with a low genetic divergence between them (Garros et al., 2014; Mathieu et al., 2011). Despite efforts to differentiate between them using species‐specific assays, complete discrimination between *C. obsoletus* and *C. montanus* remains elusive. Therefore, the accuracy of PCR assays is hindered by unresolved phylogenetic relationships and the high genetic similarity shared between the two species (Aguilar‐Vega et al., 2021; Dähn et al., 2025). *Culicoides montanus* has not been reported in Ireland, and its vector competence and public health significance in Europe are largely unstudied, so it was not prioritised in this study. However, primers designed by Goffredo et al. (2010) for *C. montanus* based on the ITS2 region could be useful in regions where *C. montanus* and *C. obsoletus* occur sympatrically.

A Ct cut‐off value of 34 was used in all assays to improve the test accuracy, ensuring good sensitivity and specificity (Caraguel et al., 2011; Greiner, 1995). This ensured that only reliable amplification signals were interpreted as positive. The cut‐off was based on primer efficiency testing, balancing the detection of low DNA concentrations and minimising false positives/negatives. For assays such as that developed for *C. obsoletus*, the application of this Ct cut‐off value effectively excluded the high Ct signals observed during cross‐species reactivity testing (Table S3), which likely reflect low‐level, non‐specific amplification or background noise. Implementing this threshold also minimises the risk of false positives when the assay is applied to mixed‐species field traps. Overall, the results indicate that cross‐species amplification is minimal and unlikely to compromise the accuracy or reliability of the assay.

When applied to field trap samples, pool sizes ranged from 21 to 96 individuals and were considered suitable for the assays, with no evidence of reduced performance. The observed detection patterns indicate broadly consistent occurrence of *C. obsoletus* and *C. chiopterus* across all sites. *C. dewulfi*, *C. pulicaris* and *C. scoticus* also appear to be widely present across sites, with only minor variation in detection. In contrast, the more variable occurrence of *C. punctatus* and *C. impunctatus* suggests potential spatial differences in habitat suitability or local population dynamics. These real‐time PCR assays provided a semi‐quantitative assessment of the number of target species in individual light trap catches, with lower Ct values indicating higher target DNA concentrations. Cêtre‐Sossah et al. (2008) emphasised that detecting higher numbers of species, like *C. imicola*, is more critical for risk assessment than low numbers of individuals. In this study, Ct values were used to estimate the relative abundance of each *Culicoides* species within the mixed‐species pools. Lower Ct values were interpreted as indicative of higher DNA concentrations and, therefore, a greater number of individuals of the corresponding species. This approach provides an approximate estimate of species prevalence in pooled samples. However, it is important to consider that interspecific variations in body size, such as the relatively larger size of *C. pulicaris* compared to other species, may influence DNA yield and thus affect Ct‐based abundance estimates. Regarding absolute quantification, we acknowledge that it may not be directly inferred from Ct values due to biological and technical sources of variation, including interspecific and intraspecific differences in body size, leading to unequal DNA contribution per individual; variation in DNA extraction efficiency and PCR amplification among species. Therefore, Ct values should be interpreted as semi‐quantitative measures of relative abundance rather than exact counts. Despite these limitations, the method represents a substantial improvement for epidemiological surveillance by enabling rapid screening of large sample numbers, consistent detection of key vector species and comparative analysis of species presence/absence across spatial and temporal scales.

In addition to enabling rapid detection of adult midges in trap samples, these species‐specific real‐time PCR assays should also be capable of detecting larvae in environmental samples. Although different species of *Culicoides* larvae are thought to occupy different niches within the same site (Kettle & Lawson, 1952), little is known about their species‐specific occupation in breeding sites and vector control strategies that focus on managing breeding sites have been shown to be largely ineffective for reducing *Culicoides* populations (Harrup et al., 2014). Conventional PCR has been used to identify larval samples (Yanase et al., 2013); however, real‐time PCR is recommended for large‐scale studies, as it is more high‐throughput, rapid and effective for identifying breeding sites in farming environments, which are usually linked to the spread of vector species and disease transmission. The assays can also be used for biodiversity monitoring by enabling precise detection of *Culicoides* species diversity across habitats in Ireland, as has been done for other species (Harrington et al., 2019; O'Meara et al., 2012, 2014; O'Neill et al., 2013). This would support fine‐scale habitat association studies and reveal ecological preferences and potential shifts in emergence patterns driven by climate change or land use changes. Overall, this molecular approach can be used to enhance the ecological understanding of *Culicoides* communities in the Palearctic region, supporting both conservation efforts and the risk management of vector‐borne diseases.

Although this method is highly efficient, designing species‐specific primers for all species that occur in Ireland was not possible because of the high number of species present. Therefore, this study focused on designing species‐specific real‐time PCR assays for the most common *Culicoides* species, as well as those most likely to drive BTV/SBV transmission in Ireland, to improve year‐round monitoring of *Culicoides* biodiversity.

## CONCLUSION

The assays developed in this study successfully detected and discriminated all seven target *Culicoides* species, providing a valuable diagnostic tool for monitoring species composition in mixed field collections. These species‐specific assays offer a high‐throughput, rapid, cost‐effective and reliable method for tracking the distribution of *C. obsoletus, C. scoticus, C. chiopterus, C. dewulfi, C. pulicaris, C. punctatus* and *C. impunctatus* adults in trap samples. Although their ability to detect *Culicoides* larvae in environmental samples has yet to be tested, this application would greatly aid targeted surveillance and control efforts. Overall, the real‐time PCR tools developed in this study can help improve our understanding of *Culicoides* biodiversity and vector‐pathogen dynamics.

## AUTHOR CONTRIBUTIONS

**Elsie Isiye:** Writing – original draft; methodology; formal analysis; data curation; visualization; validation; software; investigation; writing – review and editing. **Angela Valcarcel Olmeda:** Resources; writing – review and editing. **Thomas Curran:** Methodology; writing – review and editing; conceptualization. **David O'Neill:** Supervision; writing – review and editing; validation; software; conceptualization; investigation; methodology; formal analysis; visualization; data curation. **Theo De Waal:** Investigation; conceptualization; funding acquisition; writing – review and editing; supervision. **Gerald Barry:** Conceptualization; funding acquisition; writing – review and editing; supervision; investigation. **Aidan O'Hanlon:** Conceptualization; investigation; writing – review and editing; funding acquisition. **James O'Shaughnessy:** Conceptualization; investigation; writing – review and editing; resources; funding acquisition. **Marion England:** Resources; writing – review and editing. **Annetta Zintl:** Conceptualization; investigation; funding acquisition; writing – review and editing; project administration; resources; supervision; methodology; validation; visualization; formal analysis. **Denise O'Meara:** Conceptualization; investigation; funding acquisition; writing – review and editing; methodology; validation; visualization; formal analysis; project administration; resources; supervision.

## CONFLICT OF INTEREST STATEMENT

The authors declare no conflict of interest.

## BENEFIT‐SHARING STATEMENT

The benefits of this research are enhanced through open sharing of data and results, as outlined above. Primer and probe sequences have been published to support broader application by researchers and/or to provide a robust framework for validating suitable detection chemistries across different contexts.

## Supporting information


**Figure S1.** Standard curves for (a) *Culicoides chiopterus*, (b) *C. dewulfi*, (c) *C. impunctatus*, (d) *C. scoticus* and (e) *C. punctatus*, showing linear regression of Ct values against DNA concentration.
**Figure S2**. Melt curve analysis for each species‐specific SYBR‐based test, showing distinct melting temperature (*T*_m_) peaks corresponding to each Culicoides species target.
**Table S3**. Summary of 13 pooled DNA samples collected from 13 field sites, including DNA concentrations and corresponding Ct values for each species‐specific assay. Results confirm the presence of individual *Culicoides* species within each pooled sample based on amplification in the respective tests.
**Table S4**. Results of in silico specificity testing for each species‐specific primer, including predicted haplotypes and sequence alignments and sequences from non‐target species with potential alignment to the COBSO probe.

## Data Availability

The data that support the findings of this study are openly available in Zenodo at https://doi.org/10.5281/zenodo.20158216.
